# Role of the TLR signaling pathway in the pathogenesis of glioblastoma multiforme with an emphasis on immunotherapy

**DOI:** 10.1016/j.bbrep.2025.102149

**Published:** 2025-07-18

**Authors:** Seyedeh Elham Norollahi, Kosar Babaei, Ali Rashidy-pour, Bahman Yousefi, Rasoul Baharlou, Bahareh Farasati Far, Amir Jalali, Ali Akbar Samadani

**Affiliations:** a Cancer Research Center and Department of Immunology, Semnan University of Medical Sciences, Semnan, Iran; b Noncommunicable Diseases Research Center, Neyshabur University of Medical Sciences, Neyshabur, Iran; c Research Center of Physiology, Semnan University of Medical Sciences, Semnan, Iran; d Department of Chemistry, Iran University of Science and Technology, Tehran, 1684613114, Iran; e Department of Applied Cellular Sciences and Tissue Engineering, Langroud School of Allied Medical Sciences, Guilan University of Medical Sciences, Rasht, Iran; f Guilan Road Trauma Research Center, Trauma Institute, Guilan University of Medical Sciences, Rasht, Iran

**Keywords:** TLR signaling pathway, The pathogenesis of glioblastoma, Immunotherapy, PD-1/PD-L1

## Abstract

The most malignant brain tumor, glioblastoma multiforme (GBM), has a high mortality rate. Recently, translational elements in GBM therapy have emerged as novel therapeutic strategies in addition to conventional treatment methods. In this way, Toll-like receptor (TLR), PI3K/Akt/mTOR, MAPK/ERK, NOTCH, and other signaling pathways have recently become some of the main signaling pathways in brain tumors. The immunological reactions to brain tumors are mediated by these mechanisms. A family of proteins known as TLRs is essential to the natural defense mechanism because it can identify and react to infections and other danger signals. TLRs have dual functions in the glioma microenvironment including that they can initially activate the innate and adaptive immune responses that support antitumor activity and secondly, their activation can also contribute to tumor progression by promoting inflammation and immune evasion, as they are expressed on both immune cells and tumor cells. TLR agonists are receiving more attention in the treatment of glioma because some of them have demonstrated survival benefits in clinical studies when used in conjunction with immunotherapy, chemotherapy, radiation therapy, and immune checkpoint inhibitors. The most exciting use of TLR agonists is that they can be used as immunomodulators to avoid dose accumulation, boost the efficiency of other therapies, and, by upregulating PD-1, reinforce delayed immune checkpoint resistance against PD-1/PD-L1 inhibition. Therefore, the use of TLR agonists can lead to PD-L1 overexpression, which in turn enhances the efficacy of checkpoint inhibitors and triggers potent anticancer immune responses. In this article, we describe the function of the TLR signaling system, the cellular and molecular elements contributing to the etiology of glioblastoma multiforme, the connection between TLRs and glioma, and their significance for immunotherapy.

## Highlights

• GBM, particularly high-grade gliomas (HGG), is considered a highly aggressive tumor due to its rapid progression, poor survival outcomes, high recurrence rate, and resistance to conventional treatments.
• Glioma immunotherapy can be made more appealing by TLR signaling and its wide range of interactions.
• Through the utilization of TLR agonists to actuate PD-1/PD-L1 expression, balance TLR-expressing GSCs and resistant cells to make an antitumor microenvironment, and smother pro-tumorigenic signaling and undesirable GAM aggregation.

## Graphical abstract

**Figure:**
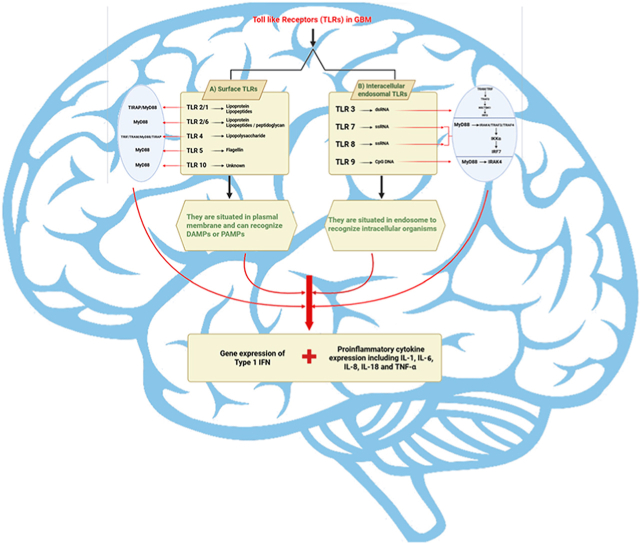

## 1 Introduction

### 1.1 TLRs and tumor-related immune responses

#### 1.1.1 TLR signaling in GBM

Toll-like receptors (TLRs) are a type of protein that are extremely important for our body's first line of defense against germs. They are mostly on immune cells, such as macrophages and dendritic cells. Their job is to identify certain patterns associated with germs (PAMPs) and signs of cell damage (DAMPs). When TLRs are activated, they generate a series of signals inside the cell that result in the production of substances like pro-inflammatory cytokines, chemokines, and other helpers that play a role in managing the immune response. In this situation, TLRs help both grow and stop GBM tumors. GBM has a lot of inflammation in its surrounding area. TLR signaling can worsen inflammation, which causes cancer cells to grow, survive, and spread more. For example, TLR-4 activation leads to an increase in substances like interleukin-6 (IL-6) and tumor necrosis factor-alpha (TNF-alpha), which can help tumor growth. In addition, GBM cells can use TLR signals to make the surroundings less helpful to the immune system [1,2]. When certain receptors in the immune system are turned on, they trigger the production of molecules like PD-L1. These molecules help tumor cells avoid being found and destroyed by the immune system. TLR signaling may have pro- or antitumor effects within the microenvironment of the tumor, depending on the kind of immune system and cancer cells infiltrating the tumor [3]. Certain TLRs are the subject of intense dispute, which can be explained by the tumor models employed in the present study. Research has found that TLR stimulation can directly affect cancer cells or trigger an immune response from other cells that helps the immune system fight tumors and leads to the death of tumor cells [4,5].

#### 1.1.2 TLR3 activation

When TLR3 is activated in different types of cancer cells, it causes the cells to die in different ways. Less survivin, stopping XIAP, FLIP, Bcl-xL, and Bcl-2, and many cells expressing the harmful caspases 3 and 8 show this [6]. It has been demonstrated that the body's defenses against MAMPs are more effective than those against tumor cells, therefore initiating the response. Studies in pharmacology have shown that when certain pathways in the body are stimulated by certain molecules, they induce the production of substances like type I interferons. These interferons can be used in therapy to modify immunotolerance and generate antitumor responses although they are not activated by tumors themselves.

#### 1.1.3 The role of dendritic cells (DCs) in GBM

Dendritic cells (DCs), an expert antigen-presenting cell type, are essential for the anticancer immune response [7,8]. Dendritic cells play a role in both the innate and adaptive immune systems by interacting with T and B cells and by expressing all TLRs. An effective immune response to malignancies requires IFNs [9]. Therefore, because IFNs remove DC-induced resistance and produce an anticancer response, activating the TLR-IFN type I signaling pathway is crucial for treatment. DCs interact with tumor cells through several mechanisms. Through phagocytosis, they can engulf apoptotic or tumor-derived extracellular vesicles containing antigens. Additionally, DCs absorb soluble antigens released by tumor cells via endocytosis. After taking up these antigens, DCs process them and present them on major histocompatibility complex (MHC) molecules. MHC class I molecules present antigens to CD8^+^ T cells, whereas MHC class II molecules present antigens to CD4^+^ T helper cells. Mature DCs activate naïve T cells upon migrating to lymph nodes by presenting processed tumor antigens. Moreover, DCs produce various cytokines that influence immune responses. For example, pro-inflammatory cytokines such as IL-12 enhance Th1 responses and increase cytotoxic T-lymphocyte (CTL) activity. Conversely, regulatory cytokines like IL-10 and TGF-beta can lead to immunosuppression if produced in excess. Furthermore, by presenting antigens and triggering a T-cell response, DCs triggered by TLRs can drive anticancer responses by causing Cell death in cancer cells [10].

#### 1.1.4 Specific TLRs and antitumor responses

It is known that DCs triggered by TLR7 ligands can cause cell lysis to trigger antitumor responses [11]. Conversely, flagellin-induced TLR5 activation can boost DC anticancer activity [12]. When the immune system kills tumor cells with the help of certain cells, it makes it easier for other immune cells to recognize and attack the tumor. TLR9-stimulated DCs likewise generate antitumor responses [13]. IFN is crucial for controlling tumor growth since it has the power to control the activities of natural killer cells (NK). Myeloid DCs can be activated by TLR3 with poly (I: C), which leads to an NK cell response and tumor shrinkage, as observed in a study using mice with melanoma. It is now known that IRF-3 is required for the NK-activating molecule to connect with myeloid DCs and NK cells [14]. Among the various TLRs involved in GBM, TLR2 has been identified as a key mediator in modulating the tumor microenvironment. It is primarily expressed on tumor-associated macrophages and microglia, where it contributes to immune suppression and supports tumor progression. Inhibition of TLR-2 signaling has been shown to reprogram the immune response toward a more pro-inflammatory and antitumor phenotype [15]. In addition, Tregs are important for the immune response and for teaching the body to tolerate certain substances. TLRs on DCs help control Treg activation by sending signals that stop IL-6 from weakening the immune system [16]. Moreover, TLR8 agonists can stimulate anticancer responses and suppress Treg activity independently of DC activity [17]. By causing tumor regression, the poly (I: C) agonist of TLR3 can change tumor macrophages into tumor suppressor macrophages, which release inflammatory cytokines (M1 macrophages). TNF-α mediates this alteration via a separate MyD88 mechanism [18]. Moreover, TLR9 agonists can inhibit angiogenesis and exert anti-cancer effects [19]. Because it inhibits angiogenesis and metastasis and decreases tumor growth, TLR-induced interferon plays a significant role [20,21]. On the other hand, tumor cells can activate TLRs, which, when combined with a particular type of tumor, can result in the proliferation, survival, or death of tumor cells, as well as chemotherapy resistance [3].

#### 1.1.5 TLR-4 vs. immune checkpoints

T-cell modulation is possible through molecules known as immune checkpoints (ICs). A balanced immune response is guaranteed by costimulatory and co-inhibitory characteristics. ICs enhance the immune system's appropriate response while safeguarding autoimmunity. Aggressive tumor invasion results from IC signaling activation as a pathway for immune evasion during carcinogenesis. Found in both types of T cells, CD-8+ and CD-4+, The first IC molecule fully described by experts is called cytotoxic T lymphocyte antigen 4 (CTLA-4). It pairs with a specific partner, B7–1, or an alternate name for it would be CD80, which is found on APCs, namely antigen-presenting cells. This pairing results primarily in the middle of these two ends up curtailing activity within our body's mighty T-cells! There is a strong association between poor prognosis and brain tissue invasion in GBM and CD8^+^ and CD4^+^ cells' expression of CTLA-4. TLR signaling increases CD80 upregulation, which improves antigen presentation. It bolsters GBM growth and T-cell anergy produced by CTLR-4 [22,23]. PD-1/PD-1L is an additional inhibitory channel. The two most powerful immunomodulatory proteins are PD-1 and PD-L1. The research group led by Dong classified PD-1L as a B7–H1 molecule in 1999 [24], which is a 20 % homology level representative of the B7 protein family. There are two types of PD-1L expression: constitutive and inducible. Resting lymphocytes and antigen-presenting cells can satisfy this requirement. Induced expression occurs in immune response and inflammation; PD-1L is a symbol of suppressive qualities. Both constitutive and induced expression levels of PD-1L are represented by glioblastoma cells [25]. There is also the autoinduction phenomenon via TLR-4. When TLR-4 is activated in GBM, signaling via the AP-1s, TRAF6/ERK-1/2, and MyD-88-independent pathways occurs, activating the PD-1L promoter. The message in the DNA that encodes PD-1L is used to encode PD-1L proteins. These proteins are then found outside the GBM cells after processing in the Golgi apparatus. T cells attach a suitable PD-1 receptor to PD-1L after recognizing it. An inhibitory cascade is triggered by a (ITSM tyrosine-based switch motif found in the intracellular region of the receptor [26]. The interaction between tyrosine phosphatase 2/zeta chain-related protein kinase 7 (SHP-2/Zap 70) and downstream signaling induces dephosphorylation and significantly reduces lymphoid cell cytotoxicity and growth. Enactment of the PD-1/PD-1L pivot raises the recurrence of lymphocyte apoptosis and T cell anergy. 2020, 21, 3114 7 of 21 Int. J. Mol. Sci. High-mobility bunch box-1 individuals of the warm stun protein (HSP) family proteins (HMGB1) and, lipopolysaccharides (LPsS) function as TLR-4 agonists, initiating a signaling cascade that promotes PD-1L production. A lot of these molecules are found in the GBM area, and this makes the PD-1L/PD-1 axis the main controller of the immune system in that area [27]. Beswick et al.'s study [28] discussed the mechanism of TLR-4/PD1L inhibition in the colonic mucosa. In addition, Wolfle et al. demonstrated a similar effect of TLR-4 activation on programmed death ligand-1 (PD-1L) expression [29]. The publication by Zhao et al. The poor prognosis of patients with peripheral lymphoma is associated with overexpression of TLR-4 and PD-1L supports the carcinogenic role of the axis [30].

#### 1.1.6 Central nervous and lymphatic systems

The brain and spinal cord were thought of as smart parts of the body. This idea is backed up by how the body works. For example, not having a normal lymphatic system and having a blood-brain barrier (BBB) can lower the body's immune responses [31]. Numerous studies have refuted this theory. The lymphatic central nervous system was discovered in 2015. Research has shown that deep cervical lymph nodes and the central nervous system are specifically connected. Through passageways filled with cerebrospinal fluid, antigens, and T lymphocytes are transported. The PD-1 protein controls the activation of T cells by interacting with APC cells like microglia, macrophages, and dendritic cells. T cells can enter the CNS to find cancer cells. Active T cells have more PD-1 receptors than other cells. Glioblastoma cells and microglia with high levels of PD-L1 help PD-L1 stick to PD-1, which stops the immune system from working properly. This negative regulation has the largest effect on T-cell responses [32]. The CNS's interaction with the lymphatic system is improved by the BBB failure, which is frequently observed in patients with GBM and the inflammatory conditions that accompany it [33]. Inhibitory cytokines are released from glioma cells to suppress local immune responses. Prostaglandins, particularly prostaglandin E2 (PGE-2) and interleukin-10, have direct effects on the proximal intercellular matrix. TGF-β limits the response of the local immune system. GBM upregulates the surface expression of immune checkpoint molecules, such as PD-L1/PD1, leading to immunosuppression [34]. There is evidence that GBM can eradicate Th1-related cytotoxicity and CD4^+^ T-cells from tumor tissues. GBM may result in long-term immune system inflammation, with Th17 cells mediating the process, which would facilitate the growth of the tumor [35].

#### 1.1.7 Anti-PD-1/PD-L1 immunotherapy

Monoclonal antibodies (mAb) have made significant advances in cancer therapy. They obstruct immune effector cells' receptors or neoplastic cells' ligands on accessory cells (moreover known as antigen-presenting cells, or APCs). The ability to block PD-L1 and PD-1 in oncological therapy has been made possible by our growing understanding of their roles in the pathophysiology of neoplasms. Many medications are presently undergoing clinical trials to treat various illnesses, including leukemia, melanoma, lymphoma, renal cell carcinoma, ovarian and stomach cancer, and lung cancer [36]. Several mAbs have received approval for clinical use from regulatory authorities in some countries [37]. Nivolumab, a monoclonal antibody of the human IgG4 class targeting PD-1, is a notable example of such agents. The mechanism of action of PD-1 involves the modulation of the immune response through inhibition of PD-1 molecules [38]. Another illustration is PEMBROLIZUMAB. This humanized mAb functions by obstructing PD-1. Both the European Drugs Office (EMA) and the U.S. The Nourishment and Medicate Organization (FDA) has allowed administrative endorsement for the clinical application of these drugs within the administration of non-small-cell lung cancer among people who have already experienced chemotherapy. Japan has also approved nivolumab [39]. It has also been demonstrated to be effective in treating melanoma, Chodkin's lymphoma, kidney cancer, and lung cancer [40]. PD-L1 blocking is a mechanism used by some medicines. This category comprises DURVALUMAB and AVELUMAB, which contain human sequences, as well as ATEZOLIZUMAB, a humanized monoclonal antibody [36]. In the context of glioblastoma, investigations are underway regarding the use of PD-1 and PD-L1 immunotherapy. Research conducted using preclinical mouse models of glioblastoma has confirmed the effectiveness and safety of monoclonal antibodies targeting PD-1 and PD-L1. Their strong anti-cancer potential is shown by the data that have been collected thus far, which include prolonged animal lifetimes and significant regression of tumor mass [41]. The trials include patients with recurrent GBM who are treated with monoclonal antibodies such as PD1 and PD-L1 inhibitors [42]. In the 2017 Reardon et al. study, known as the CheckMate 143 trial, 369 patients with glioblastoma experienced their first relapse following radiation and TMZ therapy. The study compared the efficacy of bevacizumab administered biweekly at a dose of 10 mg/kg with that of nivolumab administered biweekly at a dose of 3 mg/kg. Patients treated with nivolumab displayed a middle-by-large survival, of 9.8 months, whereas those treated with bevacizumab had a middle-by- large survival of 10 months. In the nivolumab group, the response lasted an average of 11 months, while in the bevacizumab group, it lasted only 5. 3 months. The general response frequencies were 23 % and 8 % in the bevacizumab and nivolumab cohorts, respectively [43]. Reardon et al. conducted an open-label stage 2 trial in 2017 to test durvalumab, another pharmaceutical from the bunch, in patients with GBM. Durvalumab (10 mg/kg) was used as monotherapy in this study at a dose of 10 mg/kg. The bevacizumab for repetitive GBM patients showed a halfway reaction of 13,3 %, while 46,7 % of patients in this cohort (n = 30) appeared a steady reaction. At 12 months, four patients [44] did not experience progression (Table 1). While the bevacizumab group's OS did not extend in these trials, the addition of inhibitor checkpoints to glioblastoma treatment may have various advantages. For patients with GBM, further clinical trials are needed to determine the optimal therapy and prevent adverse reactions. the optimal clinical outcome is typically achieved with the concurrent application of multiple therapeutic modalities, each based on a different range of anticancer mechanisms. In a mouse model, combined treatment involving PD-L1 inhibitors and MAPK or PI3K inhibitors produced encouraging outcomes [45,46]. Because glioblastoma has not yet been the subject of comparable observations, patients with this type of tumor may benefit from multiple-targeted immunotherapy. Anti-PD-1 and anti-PD-L1 medications cannot be used for all patients, even with encouraging research results. It has been demonstrated that 61 %–88 % of GBM patients have programmed death-ligand 1 expressed in their tumor cells [47]. Nevertheless, PD-L1 is not a dummy variable. The histological type of GBM and the range in which PD-L1 expression is expressed define the severity. PD-L1 expression affects the response of patients to PD-1 and PD-L1 treatment. Compared with individuals with low PD-L1 levels, those whose tumors expressed the protein exhibited a stronger response to anti-PD-1 therapy. These investigations, however, focused on cancers other than gliomas, such as renal cell carcinoma, lung cancer, prostate cancer, colorectal cancer, and melanoma [48]. Other studies that looked at the relationship between tumor cells' PD-L1 levels and lymphocytes' PD1 levels and clinical responses came to similar conclusions. The degree of this association was weak at the PD-1 level [49]. However, more investigation is needed to accurately understand this correlation. A large number of patients in the PD-L1-expressing category do not react well to relevant checkpoint blockade. However, it has been demonstrated that in different cancers, a subset of the research group that tested not positive for PD-L1 had favorable medical outcomes [50,51].

**Table 1:** Phase I–II clinical trials using monoclonal antibodies against PD-1/PD-L1.

Clinical trial Identification Number	Monoclonal Antibody	Number of Patients	Study Phase
NCT02829931	Nivolumab	26	I
NCT02313272	Pembrolizumab Bevacizumab	46	I
NCT02529072	Nivolumab	66	I
NCT02658981	Anti-LAG-3, Urelumab, Nivolumab	68	I
NCT02526017	Nivolumab	280	I
NCT03233152	Ipilimumab, Nivolumab	6	I
NCT02937844	Anti-PD-1 CSR T Cells	20	I
NCT03058289	Anti-PD-1 antibody	60	I//I
NCT02327078	Nivolumab, Epacadostat	291	I//I
NCT02311582	MK-3475, MRI-guided laser ablation	52	I//I
NCT02866747	Durvalumab	62	I//I
NCT02798406	Pembrolizumab	48	II
NCT02335918	Varlilumab, Nivolumab	205	II
NCT02968940	Avelumab	43	II
NCT02794883	Durvalumab Tremelimumab	36	II
NCT02336165	Bevacizumab	159	II
NCT02337491	Pembrolizumab Bevacizumab	82	II
NCT03014804	Nivolumab	30	II
NCT02550249	Nivolumab	29	II
NCT02852655	Pembrolizumab	30	Pilot

#### 1.1.8 Emerging immunotherapeutic targets in GBM

In addition to well-studied immune checkpoints such as PD-1/PD-L1 and CTLA-4, recent studies have highlighted the role of the CD200–CD200R axis in contributing to immunosuppression in glioblastoma. CD200 expression in GBM has been shown to impair antitumor immunity by engaging inhibitory receptors on immune cells. Targeting CD200 in combination with other immune checkpoint inhibitors or TLR agonists represents a novel strategy under current investigation [52].

#### 1.1.9 Immunotherapy focusing on TLR activation

**Fig. 1:**
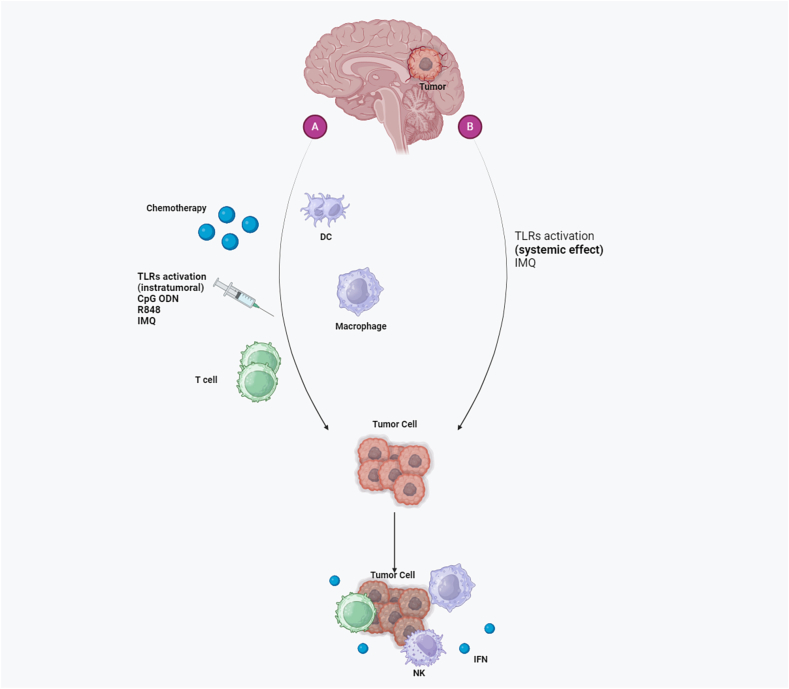
Overview of the immunotherapy pathways of TLR agonists targeting CNS malignancies. TLR activation triggers the release of cytokines, as well as the death of tumor cells, dendritic cells, macrophages, T cells, and active natural killer (NK) cells. TLR agonists can have two types of effects: systemic (B): These involve boosting the immune system across the body, which improves the body's ability to fight tumors. Systemic effects can cause the body to produce more pro-inflammatory cytokines and activate different immune cells. This helps fight tumors in areas beyond where the treatment was given [80]. and local (A): These are limited to where the TLR agonist is given. Local activation can change the area around a tumor, causing inflammation and the entry of immune cells into the tumor. This targeted approach can make other treatments, such as chemotherapy or radiation, work better by making tumor cells more responsive to them [81].

Therapeutic targets for the treatment of many malignancies, including TLR agonists, have been proposed [3]. Many artificial ligands are being researched for immunotherapy applications. The most widely utilized TLR agonists in therapy are ODNs, which stimulate the production of cytokines and activate TLR responses in antitumor T cells, dendritic cells, monocytes, and innate and adaptive immunity [53]. When ODN 1826 stimulates TLR9, gliomas undergo caspase-3-dependent apoptosis, extending the lifetime of C57BL/6 mice with an intracranial glioma cell line (GL261). In addition, ODN 2138-treated mice did not exhibit any signs of improved survival [54]. Furthermore, ODNs improve long-term immunity and longevity in mice harboring two distinct types of glioblastoma tumors, of which only one was treated. These findings imply that CpG-ODN treatment could be effective for tumor cells that are situated some distance from the application site. The antitumoral effect was mediated by resistant system cells, cells like NK cells, macrophages, microglial cells, and CD8 T cells, instead of directly causing harm [55]. TLR5, TLR7, and TLR9 are present in GL261 cells at low to undetectable levels, according to a different study by Grauer et al. Surprisingly, a single intratumoral injection of CpG ODN 1668 in C57BL/6 mice suppressed glioma development and cell proliferation in a cell-type-specific way. Compared with PAM3CysSK4 (median survival = 34.5 days), CpG ODN 1668 was more effective in eliminating murine gliomas (median survival >90 days), although LPS and poly (I: C) did not significantly affect tumor growth (median survival = 27 days). R848 increased glioma-bearing mice's survival like ODN 1668, although less significantly (median survival >36.5 days) [55]. A cure rate of 55 % was achieved by subcutaneously immunizing the survival of glioma-bearing mice with CpG-ODN 2006 glioma cell lysate (cell line GL261). The mice exhibited a significant increase in activated DCs and a notable expansion of T lymphocytes, which produced IFN-α and lysed glioma cells. These results are consistent with the notion that intratumoral CpG ODN administration is inferior to extracranial priming of T cells with CpG-activated DCs expressing tumor antigens [56,57]. The authors report that this approach is safer, easier to use, and more effective for delivering CpG ODN during glioma immunotherapy. Further research is necessary because cell-line gliomas are known to be more immunogenic than gliomas that develop in humans [58]. Carbon nanotubes (CNTs) have been investigated as a thiolate CpG (sCpG) delivery vehicle into tumor-associated immune cells in a mouse glioma model. This enhances the CpG ODN effect by promoting internalization to target cells. In mice with pre-existing gliomas, 50 %–60 % of them were cured when CNT-sCpG slowed tumor growth. This medicine helped reduce cancer, and caused, more natural killer cells in the blood and macrophages in the brain. Although sCpG alone increased mouse survival, the effect was not as great as that when CNT-sCpG was administered to the mice [59]. The potential systemic effect of TLR activation is another benefit of TLR agonists in immunotherapy. The number of DCs and tumor-reactive T lymphocytes that reached the glioma site was significantly boosted by the topical application of IMQ, as revealed by Xiong et al. Furthermore, as the tumor cells did not produce TLR7 mRNA, soluble IMQ reduced the growth of GL261 cells in a TLR7-independent manner [60,61]. The adenosine receptor-mediated signaling pathway may be responsible for IMQ's ability to limit tumor growth because its inhibitory effects on glioma cells are independent of TLR7 expression [60]. TLR7/TLR8 are not expressed in the CNS-1 rat model of glioma, according to a different study [61]. Nevertheless, TLR7/8 activation by R848 alone was sufficient to reject the smaller developed tumor in CNS-1. In a glioblastoma model, intratumorally injected LPS caused a 50 % reduction in TLR4 deficient animals and nearly total subcutaneous tumor removal in wild-type BALB/c mice. Nevertheless, in mice with cerebral glioblastomas, this treatment did not provide significant benefits. The tumors removed from wild-type mice did not express TLR4. Nonetheless, both cancers have neutrophilic and macrophage-rich immune systems. There is evidence to suggest that TLR4 is not the sole mediator of the immunity-related antitumor effect of LPS. Taken together, these results indicate the involvement of stromal and immune elements in the tumor microenvironment. It is important to carefully consider the use of LPS in the treatment of CNS malignancies, as some data have indicated that this TLR4 agonist has neurotoxic and inflammatory effects. As a result, less toxic alternatives have been suggested [62,63]. According to Kawanishi et al., in C3H/HeJ mice, Spirulina complex polysaccharides (CPS) caused a higher generation of IL-17 and started an antitumoral response against glioma compared with LPS; however, the outcome was different for C3H/HeN mice. The findings confirmed that TLR4 signaling is necessary for these effects. In each animal strain (C3J/HeN and C3J/HeJ), anti-IL-17 antibodies decreased the proliferation of glioma cells, but they did not affect the growth inhibition caused by Spirulina CPS in C3J/HeN mice. Furthermore, CPS-treated C3H/HeN mice displayed low levels of CD31 (angiogenesis marker), acquired immunity, and decreased IL-17 concentrations. Ultimately, we determined that Spirulina CPS-TLR4 signaling facilitated the inhibition of glioma growth in T cells, macrophages, and natural killer cells. The scientists deduced that CPS's antitumoral effect stems from its capacity to reduce angiogenesis and, to a lesser extent, control IL-17. They also showed that IL-17 and IFN-α production stimulates the antitumor effect of E. coli LPS, but LPS did not affect glioma angiogenesis. However, additional research revealed that the spirulina CPS compound might activate NF-κB through TLR2 and TLR4. These results imply that Spirulina CPS can exert its effects through pathways other than TLR4 [64,65]. According to a different study, TLR4 deficiency prevented the growth of U87 tumor xenografts. Moreover, TLR4 gene deficiency produced a caspase-3-dependent apoptotic mechanism, that inhibited tumor growth. Accordingly, TLR4 may be a useful biomarker for tumor prognosis and metastasis [66,67]. However, evidence of LPS's anticancer effect has been presented. LPS-TLR4 activation, according to Hua et al., LPS-TLR4 activation increases glioma development and reduces mice survival, but it does not increase proliferation in vitro. Additionally, glial fibrillary acidic protein (GFAP) was dose-dependently downregulated by this activation. MAPKs, ERK, JNK, and p38 were somewhat phosphorylated upon exposure to LPS, whereas NF-κB phosphorylation and the NOTCH pathway, which is dependent on MyD88, were significantly elevated. The suppression of GFAP expression was restored by inhibiting the Notch pathway, suggesting that the LPS-induced reversal of glioma differentiation is mediated through the Notch signaling cascade, which is dependent on MyD88 [68]. Notwithstanding these findings, there has been debate over TLR2's involvement in the CNS’ anticancer responses. In a mouse glioma model called GL261, TLR2 activation with a synthetic bacterial lipoprotein in conjunction with tumor antigen-specific CD8 T cells improved long-term survival and immunological memory [69]. On the other hand, TLR2's protumorigenic ability was also demonstrated. When TLR2 was absent from mice implanted with GL261 glioma cells, the tumors were significantly smaller, the expression of membrane type 1 matrix metalloprotease (MT1-MMP) was decreased, and the survival rates were higher than in wild-type control mice. Pam3CSK4 and MALP2, two TLR2 antagonists, cause an increase in MT1-MMP expression, which aids in the growth and progression of gliomas [70]. Nevertheless, an atypical disruption in the tyrosine phosphorylation of STAT1 induced by a TLR2 ligand has been observed in cancerous cells, rather than in healthy glial cells. The levels of tyrosine phosphorylation in STAT1 increased in microglia, astrocytes, and neuroblastoma cells exposed to LTA and Pam3CSK4, which are two TLR2 ligands. Conversely, this phenomenon was not detected in neuroblastoma cells or specific glioma cell lines (GL26, U87, and U373) [71]. Furthermore, some endogenous ligands, such as DAMPs, may have therapeutic value. Curtin et al. developed immunotherapy by infusing thymidine kinase (TK) and the Fms-like tyrosine kinase 3 ligand (Flt3L) into glioblastomas through adenoviral vectors. TK is a conditional cytotoxic gene; Flt3L causes DC infiltration into the brain parenchyma. Subsequently, scientists discovered that HMGB1, a natural activator of TLR2, with high mobility group box 1 secreted by tumor cells upon death. Regression of Flt3L/TK-induced glioma brain tumors is hindered when HMGB1 was suppressed. HMGB1 generated from tumor tissues activates CD8^+^ T cells against glioblastoma and initiates TLR2 signaling [72]. However, HMGB1 does not exclusively act as a ligand for TLR2; it can also interact with TLR4, TLR9, and RAGE receptors. It can also initiate several signaling pathways, including NF-κB, ERK1/2, p38, and STAT3. These pathways then govern the regulation of adhesion molecules, cytokines, chemokines, chemokines, migratory cells, differentiation, phagocytosis, autophagy, and tumorigenesis [73,74]. These results show that both TLR signaling and subsequent responses are quite variable in CNS malignancies. Additionally, there is evidence that NF-κB can activate on its own without the help of TLRs. In glioblastoma multiforme, NF-κB activity has been linked to carcinogenesis [75]. However, TNFα◽-induced NF-κB activation in glioma cell lines (A172 and LN229) involves both MyD88 and TRIF and is partially dependent on TLR4 [76]. However, the results obtained thus far are contentious and have not led to a conclusive position concerning the use of TLR agonists as adjuvant treatment for CNS malignancies. Several clinical-phase studies have been conducted, and others are presently underway. Clinical trials in phase I have been conducted to determine the safety profile of CpG-28 in individuals with recurrent glioblastoma. Patients received CpG-28 treatments at escalating dosages and underwent evaluation for, at least 4 months. In the largest perpendicular diameters, two patients experienced tumor reductions of 29 % and 20 %, which were linked to a decrease in the mass effect and edema in the surrounding area. The conditions of two other patients remained constant for longer than four months. Twenty percent of the patients (n = 24) had passed away at the time of the antitumor response study, and twenty-eight percent had survived for one year; the median survival was 7.2 months. In summary, phase I trials and preclinical models have shown that local administration of CpG ODN is feasible and well tolerated at doses up to 20 mg in patients with glioblastoma who are also undergoing recurrent GBM [77]. Therefore, CpG28 was given to each patient in a phase I trial by Ursu et al. that involved patients with various cancer forms, such as glioma (n = 1), ependymoma (n = 1), oligodendroglioma (n = 1), oligoastrocytoma (n = 1), and glioblastoma (n = 15). Patients may have undergone CpG therapy in addition to or instead of cancer treatment in certain cases. The findings revealed variations among the patients (n = 29). There were no notable disparities in survival rates between the cohorts administered with CpG-28 alone and those receiving a combination of CpG and chemotherapy. Three patients, nevertheless, displayed notable modifications. The patient with grade III ependymoma was. After receiving CpG28/bevacizumab therapy, patients diagnosed with grade III anaplastic oligoastrocytoma and glioblastoma showed clinical improvement, maintained stability, and succumbed at 12.5 months and 8.8 months, respectively [78]. As an adjuvant after surgical resection with standard chemoradiotherapy, immunization with autologous DC pulsed with glioma tumor lysate was found to be safe in another phase I research because it did not cause dose-limiting toxicities. Furthermore, because 5 % imiquimod, poly-ICLC, or innate immune response modifiers (TLR agonists) may enhance DC activation and T-cell priming, the study authors employed “boost” vaccines that exhibited no extra toxicity or side effects. Patients who were vaccinated demonstrated a median survival period of 31.4 months, whereas those with glioblastoma who underwent resection and concurrent chemoradiotherapy exhibited a median survival duration of 18.6 months [79] (Fig. 1). In individuals with recurrent glioblastoma, a phase II trial was conducted to assess the safety and tolerability of CpG ODN (10 mg/mL). Although the study included several long-term survivors, the investigators found no progression-free survival in any patient they studied. This suggests that some people may benefit from this treatment. To confirm whether adverse effects were caused by CpG ODN treatment and to identify patient subgroups that benefit from the treatment, additional research with a larger patient population is necessary [77]. To further elucidate the impact of immunotherapy targeting TLR activation in CNS malignancies, more patient-centered trials are needed.

**Fig. 2:**
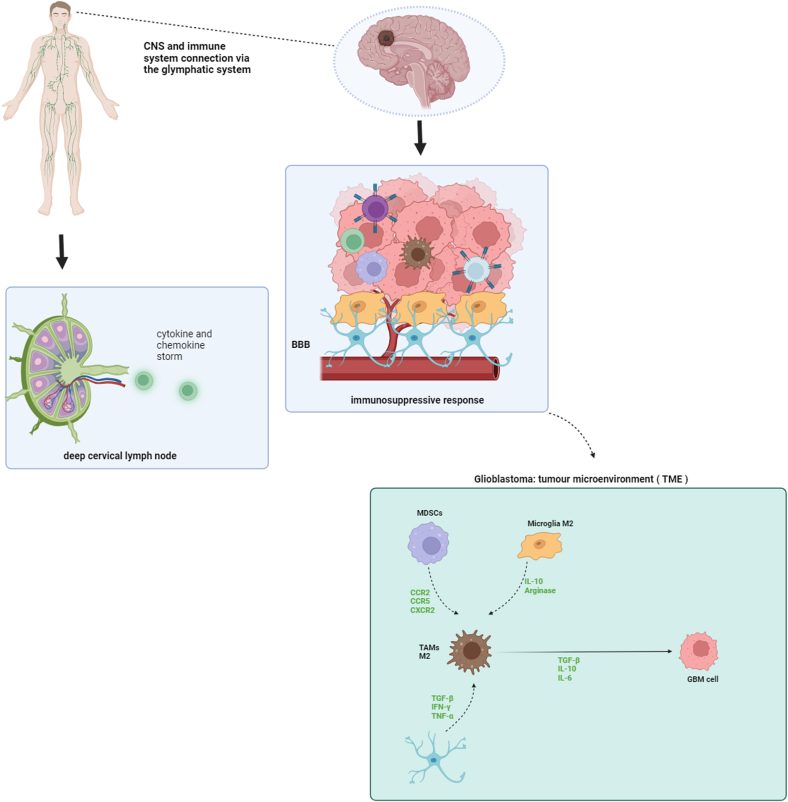
Alterations in the glioblastoma tumor microenvironment and their correlation with the peripheral immune system. The immune system of GBM consists of different types of cells, including microglia, special macrophages connected to glioma (GAMs), T cells, and natural killer (NK) cells. These immune cells can make up to half of the tumor size and greatly affect how the immune system fights the tumor. However, factors that weaken the immune system, found in the area around tumors (called the tumor microenvironment), like certain proteins known as IL-10 and TGF-β, create a very strong environment that makes it difficult for the body to fight cancer. This blockade is important for tumor growth and makes immunotherapy harder [97].

Immune Microenvironment for GBM The cells that make up a tumor microenvironment (TME), including endothelial, immunological, and other parenchymal cells, have an impact on the intrinsic molecular and genetic alterations of GBM. These cells not only support angiogenesis, invasion, and proliferation but also transmit immune-suppressive functions [82]. Because of the intact BBB and lack of a traditional lymphatic system to transport CNS-specific antigens to peripheral lymph nodes, the central anxious framework (CNS) has been seen for decades as a “resistant advantaged” organ [83]. Glymphatic System's Function in Central Nervous System Immune Surveillance According to recent reports, the immune system and the digestive system. can interchange fluids, macromolecules, and immune cells when the glymphatic system is functioning properly [84]. Louveau et al. discovered that there are specialized vessels in the body that look and act like lymphatic vessels, and they are found along the dural venous sinuses [85]. Via the cribriform plate, base of the skull foramina, nasal mucosa, and cervical lymph nodes, these lymphatic vessels can carry CNS antigens, T cells, B cells, and DCs, as well as cerebrospinal fluid (CSF)), into the peripheral immune system [84,86]. It is also important to emphasize that the BBB, astrocytes, and microglia comprise a complex network of interactions that carefully regulate immune cell migration to the central nervous system [87]. The pro-inflammatory condition that follows GBM carcinogenesis may further weaken the BBB's integrity by increasing its permeability and allowing lymphocytes and monocytes from the peripheral lymphatic system to infiltrate the bloodstream.28 In addition to being an essential part of the blood-brain barrier, astrocyte cells have the ability to increase proinflammatory activity by secreting cytokines such as TNF-α, IFN-α, and TGF-β (transforming growth factor-β) [88]. Landscape of the Immunosuppressive GBM Microenvironment The GBM TME does, in fact, contain a diverse range of immune cell types, Some cells in the body can weaken the immune system, like MDSCs, microglia, TAMs, Tregs, and APCs such as DCs and macrophages derived from bone marrow (BMDM) are the predominant cell types in this context [89,90]. Furthermore, the current generation of CD4^+^ and CD8^+^ T cells is often fatigued, inactivated, or functionally inadequate. These cells often have markers that help the immune system work better, such as TIM-3, LAG3, and PD-1 [90]. Large volumes of immunosuppressive cytokines, including interleukins (such as IL-6, IL-10, and TGF-β), are known to be released by TAMs. These cytokines block T cells from activating, which in turn inhibits the anti-tumoral responses of cytotoxic CD8^+^ T cells and natural killer (NK) cells [91,92]. In a similar vein, Tregs within the TME may limit the immunological response by preventing CD8^+^ T-cell accumulation. When pro-inflammatory cytokines like IL-6, TNF, and IFN-I are released at the same time, they can affect the immune system. MDSCs are immature cells that help suppress the immune system. They express the CCR2, CCR5, and CXCR2 chemokine receptors, which are then mobilized to the blood and tumor by chemokine ligands (CCL2, CCL5, CXCL1-8) and other inflammatory mediators like prostaglandin E2 (PGE2) generated by TME, VEGF, TGF-β, TNF-α, and IL-1β, IL-6, and IL-10. Additionally, TAM recruitment is influenced by the presence of CCR2 and CXCR3, and TAM polarization is influenced by the signaling of these chemokine receptors [93]. TAMs with an M1/M2 polarity exhibit a unique phenotype and produce pro- and anti-inflammatory cytokines differently. Inflammatory cytokines such as IL-2, IL-12, IFN-γ, and TNF-α are associated with the promotion of the M1-like phenotype, whereas IL-4, IL-10, and TGF-β are associated with the promotion of the M2 phenotype [94]. Microglial cells exhibit a phenotypic hallmark that is dependent on polarization [95]. TNF-α, IL-12, IL-23, and IL-1β cytokines are enhanced in microglia cells with the M1-like phenotype, according to multiple investigations. On the other hand, microglial cells exhibiting an M2-like phenotype were observed to have elevated levels of arginase protein and IL-10 expression [87,94]. Repolarization or reprogramming of microglia and macrophage cells exhibiting characteristics similar to those of M2 polarization may lead to notable improvements in the prognosis of GBM and suppression of tumor development because these cells exhibit increased resistance to treatment [96]. The association between the TME, BBB, and their interaction with the fringe safe framework is outlined in Fig. 2.

#### 1.1.10 Potential new checkpoint blockade targets and immune checkpoint inhibitors

**Fig. 3:**
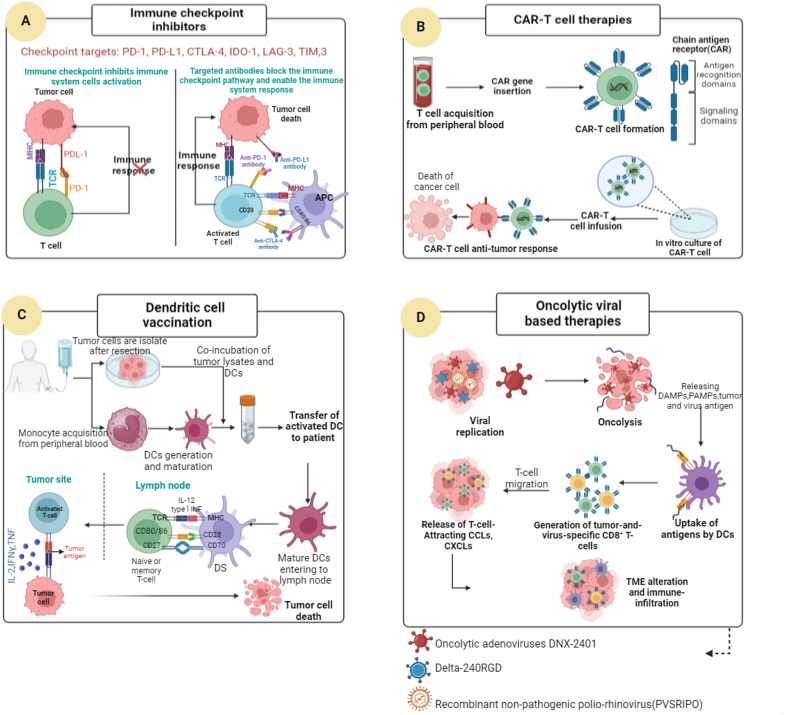
The most widely used glioblastoma immunotherapeutic approaches. (A) Immune checkpoint inhibition mechanism. (B) The development of CAR-T cells and their immediate anti-tumor activities in the context of CAR-T therapy. (C) Tumor-associated antigen peptide vaccination and autologous dendritic cell transfer after exposure to tumor lysate are two distinct vaccination approaches for glioblastoma. (D) Mechanisms of oncolytic viral therapy: from oncolysis to changes in the tumor microenvironment.

Immunoregulatory drugs that obstruct immune checkpoint molecules have recently emerged as a potentially effective therapeutic approach for several malignancies, such as head and neck cancer, lung cancer, and melanoma [98]. Due to the extremely immunosuppressive TME of GBMs, immune checkpoint inhibitors (ICPIs) have limited therapeutic benefits in patients with GBM. Although they comprise a minor portion of the TME, dendritic cells (DCs) have a significant antitumor role because of their potent ability to stimulate T-cell immunity and immunotherapeutic responses [99]. Normally, type I interferon (IFN-α) and both innate and adaptive immune responses are stimulated by plasmacytoid dendritic cells (pDCs). In addition, pDCs can function as antigens by presenting cells to control immune reactions to different antigens [100,101]. On the other hand, pDCs in cancer contribute to the development of an immunosuppressive TME by displaying a decreased capacity to react to TLR7/9, enabling decreased production of IFN-α. DC differentiation is induced by TLR signaling, which also influences interaction with T cells. TLR activation induces many biological changes in DCs, such as increased endocytic activity induced by the membrane vesicle system, cytoskeletal organization, and effects on antigen presentation through the method of protein translation and reduction. These changes enhance the quantity, quality, and perceivability of peptide-MHC complex arrangement in developing DCs [102]. TLR ligands invigorate the translation of cytokines and costimulatory components in DCs. DC maturation is controlled by signaling that occurs downstream of TLR. When TLRs are activated, DC metabolism changes from aerobic glycolytic metabolism—which is akin to Warburg metabolism in cancer cells to mitochondrial OXPHOS, which is driven by β-oxidation of fats. This metabolism, which is necessary for DC maturation, is reliant on the phosphatidylinositol 3-kinase (P13K)/Akt pathway and is inhibited by AMP-activated protein kinase (AMPK) and the anti-inflammatory cytokine IL-10 [103]. Furthermore, in vitro tests have demonstrated that mature DCs may efficiently stimulate TCR-transduced T cells by increasing the production of IL-12p70 when combined with IFN-γ and three TLR ligands, namely Resiquimod (R848), poly I: C, and LPS [104]. There are only two TLR3-expressing tumor sites: CD11b + cDC2 and CD103+ cDC1. Through the TLR3-TICAM-1 pathway, DCs generate the Th1-type cytokines IFN-β and IL-12, which aid in the development of antitumor immunity in functional cytotoxic T lymphocytes. Therefore, by focusing on TLR3 signaling in DCs, tumor-specific cytotoxic T cells can be produced [105]. A comparison between the cytokine profiles (IL-4, IL-6, IL-10, IL-12, and IL-23) in DCs stimulated by a single TLR agonist and those generated by several TLR agonists revealed that different TLR agonists had synergistic effects on boosting cytokine production. When analyzed in conjunction with other cellular signaling pathways, the p38 MAPK and ERK signaling pathways were embroiled in DCs creating IL-12p40 and 12p70 as a result of TLR signaling; on the other hand, the JNK pathway had a negative administrative impact on DCs creating cytokines when invigorated by particular TLR agonists; NF-Î°B and P13K worked as positive controllers when DCs creating cytokines as a result of TLR signaling [106]. Normal executioner cells Imperative inalienable cytotoxic lymphocytes known as common executioner (NK) cells have the fast and exact capacity to distinguish and dispense with tumor cells. By producing IFN-γ, NK cells directly control T-cell activity. They also influence the response of DCs to cancer by promoting DC maturation and attracting DCs to the TME. In the TME, several soluble cytokines control NK cells. After attachment to their appropriate receptors, cytokines such as IL-2, IL-12, IL-15, IL-18, and IL-21 improve NK cell activation, survival, proliferation, and maturation. Adoptive transfer therapy, NK cell recruitment to the TME, blocking inhibitory receptors that restrict NK cell function, and therapeutic TME regulation to boost anticancer NK cell function are among the strategies currently under investigation [107]. The use of CAR-modified NK cells (CAR-NK) may be less risky than CAR-T cell therapy, and the outcomes of adoptive transfer of autologous or allogeneic-activated NK cells for the treatment of hematologic malignancies are encouraging. However, solid tumors are more challenging because it is difficult to move and infiltrate NK cells into the tumor site, and TME inhibits NK cell activity. NK cell proliferation and antitumor activity are reduced by the release of different immunosuppressive substances by tumor cells, including prostaglandin E2, indoleamine 2,3-dioxygenase (IDO), IL-10, TGF-β and vascular endothelial growth factor (VEGF) [108]. In this way, the most widely used glioblastoma immunotherapeutic approaches, including immune checkpoint inhibition mechanism, advancement of CAR-T cells, and prompt anti-tumor exercises within the setting of CAR-T treatment, tumor-associated antigen peptide vaccination, and autologous dendritic cell transfer after exposure to tumor lysate, are two distinct vaccination approaches for glioblastoma. The mechanisms of oncolytic viral therapy are illustrated in Fig. 3.

TLRs in NK cells initiate intrinsic resistance reactions against bacterial and viral diseases by activating NK cytotoxicity and cytokine generation [109]. Different endosomal TLRs expressed by bacteria and viruses cause NK cells to express them. On CD56brightCD16 and CD56dimCD16+ NK cell subsets, all four endosomal TLRs (TLR3, TLR7/8, and TLR9) are uniformly expressed. However, the ligands for TLR7/8 (R848), TLR3 (Poly I: C), and TLR9 (ODN2395) only support NK cell function when cytokines, such as interleukins (IL-2, IL-12, IL-15, and IL-18), are present. Among them, R848 primarily stimulates cytokine production, cell proliferation, and cytotoxic action to activate CD56brightCD16− NK cells. R848 usually activates TLR7 and TLR8 on DCs, macrophages, and neutrophils; however, it exclusively engages TLR8 to activate the CD56brightCD16− NK cell subpopulation. This suggests that TLR8-targeted infiltrating TME-NK cells may play a role in the development of new tumor immunotherapies [109]. Macrophages linked with tumors The most widely invaded immune cells in the TME are tumor-associated macrophages [110]. There are two distinct classifications of macrophages: the M1 type, which is characterized by conventional activation, and the M2 type, which is characterized by alternate activation, which exhibits pro- and anti-inflammatory responses to various stimuli. The M1 macrophages exhibit pro-inflammatory characteristics when stimulated by LPS or IFN-γ, whereas the M2 macrophages exhibit anti-inflammatory properties when stimulated by IL-4, IL-13, or IL-10. In numerous cancer types, tumor-associated macrophages commonly display an immunosuppressive M2-like phenotype, promoting angiogenesis, immunosuppression, and cancer cell proliferation to facilitate tumor progression and metastasis [111]. Macrophages are partially polarized by TLR-triggered early immunological responses [112]. Chemotherapy can dramatically lower a patient's total MDSC count, but these cells still have a considerable T-cell suppressive capacity. Furthermore, STAT3 signaling and elevated IDO and IL-10 production are immunosuppressive, mechanisms [113]. By encouraging the development of monocyte MDSCs into M1-type macrophages to reduce their inhibitory function, TLR1/TLR2 signaling improves anticancer immunotherapy [114]. Oxaliplatin, a chemotherapeutic drug, suppresses the immune system by preventing MDSCs from differentiating into M1-like macrophages. By polarizing MDSCs to the M1 phenotype, however, the TLR 7/8 agonist R848 reversed the effects of oxaliplatin resistance. Oxaliplatin and R848 together increased M1-like macrophage counts and inhibited tumor growth, indicating that R848's remodeling action on MDSCs reversed the immunosuppressive effects of oxaliplatin [115]. In the meantime, R848 controls the expression of the macrophage migratory inhibitory factor (MIF) in several organs and cells [116]. Through polymorphonuclear MDSC proliferation and activation mediated by LIF (an IL-6 type cytokine and STAT3 activator), TLR9 expression in prostate cancer cells facilitates immune evasion. Thus, TLR9/LIF/STAT3 signaling-targeting oligonucleotide-based inhibitors (e.g., CpG-STAT3dODN) may present novel prospects for prostate cancer immunotherapy [117]. TLR-mediated M1 polarization is inhibited by tumor-associated macrophage activation [118]. Tumor-associated macrophages produced from blood upregulate immunosuppressive cytokines in human gliomas and are linked to a markedly reduced survival rate in low-grade gliomas [119]. Resistin influences chemotherapy resistance and the advancement of pancreatic cancer. It is released by macrophages in the TME and is regulated through its receptors, CAP1 and TLR4 [120]. The resistin effect also stimulates the TLR4/Src/EGFR/PI3K/NF-κB pathway, which in turn leads to lung cancer metastasis [121]. These biological connections within the tumor can be disrupted by nanoparticles through phenotypic changes in tumor-associated macrophages. Following systemic injection, various nanoparticles aggregate preferentially in macrophages. Macrophages identify nanoparticles as foreign substances and phagocytose or endocytose them. Several variables, including the dosage, mode of administration, size, composition, and surface characteristics of the nanoparticles, may affect the macrophage response to nanoparticles [122]. For instance, a novel PAMP mimic called acGM-1.8, a glucomannan polysaccharide with 1.8-degree acetyl alteration, selectively activates TLR2, causing macrophages to adopt antitumor behavior. Compared with earlier TLR2 agonists, this polysaccharide exhibits a better safety profile in mice studies [123]. Drugs were efficiently and selectively delivered to M2-like macrophages, M1-like macrophages, and DCs using targeted M2-like macrophage nanoparticles. To reprogram M2-like macrophages, the design uses the peptide sequence M2pep′s targeting capability to effectively and selectively deliver R848 to tumor-supporting M2-like macrophages [124]. T lymphocyte Studies involving the transfer of adoptive cells have shown that TLR is a crucial co-stimulatory and regulatory molecule in T cells. TLR agonists interact with T cells to lower the threshold for TCR activation, increase T cell production and proliferation, encourage the maturation of memory T cells, and modify Treg cells' suppressive function [112,125]. Human Treg cells suppress cellular senescence and enhance glucose intake. In a melanoma-adoptive T cell transfer therapy paradigm, TLR8 signaling specifically suppresses glucose uptake and glycolysis in Treg cells, reversing Treg suppression and improving in vivo antitumor immunity [126,127]. By upregulating CD69 and CD44 and increasing cell activation, TLR agonists additionally, contribute to the regulation of the motility of Treg cells [128]. TLR2 signaling enhances CAR-T cell antitumor efficacy. 1928zT2 and m28zT2 were created by introducing the Toll/interleukin-1 receptor domain of TLR2 into CARs that target CD19 (1928z) and mesothelin (m28z). Therefore, in vitro and in vivo, T cells expressing 1928zT2 or m28zT2 exhibit increased cell proliferation, persistence, and effects against CD19^+^ leukemia or mesothelin + solid tumors [129]. Imiquimod, a TLR7/8 agonist, stimulates natural killer cells to eliminate tumor cells, which release tumor antigens and stimulate CD4^+^ T cells specific to the tumor. Simultaneously, imiquimod causes peripheral lymphocytes to express CXCR3, a homologous chemokine receptor that causes CD4^+^ T cells to infiltrate and accumulate within the tumor and is crucial for tumor rejection [130]. Mast cells are a special subset of myeloid immune cells that reside in tissues. Mast cells can behave in three different ways [1]: directly present antigens [2]; control DC migration and effector T-cell initiation efficiency; and [3] recruit subsets of effector T cells to infected or inflammatory areas and activate homing T cells in situ to trigger an efficient inflammatory response [131]. In common, they can modify the tumor microenvironment to induce antitumor activity. In addition, the antimicrobial peptide LL-37 created from myeloid cells fortifies the arrangement of lung cancer by causing glycogen synthase kinase 3β actuation and protein kinase B, which are interceded by TLR4 in lung tumor cells and actuate Wnt/β -linked protein signaling [132]. In other situations, regulation of long-stranded noncoding RNA by TLR4 signaling may encourage the growth and malignant transformation of hepatic progenitor cells (HPCs) [133].

#### 1.1.11 Tumorigenic potential of inflammatory molecules in gliomas

Tumor rot factor-alpha (TNF- α±), interleukin (IL)-1α ± and 1β, IL-4, IL-6, IL-8, chemokines (e.g., CXCL-12), cyclooxygenase-2 (COX2), prostaglandin (PG) E2, and platelet-derived development calculate (PDGF) are among the variables determined from gliomas that are vital incendiary go-betweens that start the incendiary cycle in GBM and advance empower carcinogenesis by escaping development concealment, advancing angiogenesis and metastasis, upsetting apoptosis, and protecting the stemness of cancer cells [134,135]. It was shown that the glioblastoma cell lines CCF3 and U87-MG overexpressed IL-1α and 1β. [136]. In glioma cells, IL1β increased the pro-inflammatory prostaglandin COX-2's mRNA expression [137]. By preserving glioma cell stemness and the inflammatory milieu, Glioma aggressiveness is increased by COX-2 and its product (PG) E2, which interact with four GPCRs (PGE2 receptors like EP1, EP2, EP3, and EP4) [138]. The NF-κB pathway is activated when IL-1β binds to the IL-1 receptor, which continuously stimulates the genes that cause inflammation [139]. According to an in vitro study, VEGF is released when IL-6 is present, activating the JAK/STAT3 axis and increasing angiogenesis and cell invasion [140]. Ras, an oncogene, was discovered to interact with TNFα/IL-1β in GBM cell lines U-251 and U-87 to induce hypersecretion of IL-6/IL-8 cytokines and activate the p38 MAPK signaling pathway. This resulted in the development of an inflammatory milieu that was favorable for the advancement of cancer [141]. According to immunohistochemical research, IL-8 levels were strongly associated with 65 % of primary and recurrent GBM samples [142]. Matrix metalloproteinases (MMPs), which are powerful inducers of angiogenesis, are further stimulated by IL-8, which also controls endothelial cell migration [143]. Glioma cell lines (U87, SHG-44, and CHG-5) and human primary glioma specimens express higher levels of CXCL-12 and CXCR4 mRNA. Furthermore, the PI3K/Akt signaling pathway was activated by chemokine (CXCL-12) to induce CD133+ glioma stem-like cells to produce more vascular endothelial growth factor (VEGF), thereby increasing angiogenesis and metastasis [144]. Platelet-derived growth factor (PDGF) is frequently overexpressed in GBM and is important in the development of cancer [145]. 33 % of recurrent GBM tissues have increased expression of the receptor PDGFRα [146]. Additionally, a deviation in PDGF signaling stimulates the NF-κB, PI3K-Akt, and MAPK-ERK axis, which are critical for cell invasion, proliferation, and resistance to apoptosis [147].

#### 1.1.12 Function of tumor-associated macrophages (TAMs) in the development and spread of gliomas

Macrophages and monocytes are members of the leukocyte family, which arise from bone marrow and circulate throughout the body. Blood-circulating monocytes enter inflammatory and homeostasis-affected tissues, where they are exposed to pro-inflammatory cytokines, chemotactic chemicals, and microbial infection, ultimately differentiating into macrophages [148]. In a cytokine-rich tumor environment, macrophages can polarize into classically activated (M1, anti-tumor) or activated (M2, pro-tumor) macrophages. M2-type macrophages are stimulated by IL-4 and IL-13, whereas M1-type macrophages are stimulated by lipopolysaccharide (LPS) or PAMPs, IFN-γ, and GM-CSF [148]. Tissue-resident microglia and blood-circulating monocytes make up the majority of the infiltrating immune cells in GBM, with TAMs making up approximately 30 %–50 % of them [149]. According to previous reports, macrophages M1 and M2 have proinflammatory and immunosuppressive effects on gliomas, respectively [150]. It has been demonstrated that derived macrophages and tissue-resident macrophages (microglia) share both M1 and M2 phenotypes, which are polarized in response to distinct stimuli [151]. Actuated M1 macrophages deliver pro-inflammatory cytokines (TNF-α ±, IL-1, IL-6, IL-12, IFN-γ, IL-23), chemokines (CXCL9, CXCL10, CXCL11, CXCL16, CCL2, CCL3, CCL5), responsive oxygen/nitrogen species, and COX-2 in expansion to their cytotoxicity against organisms (e.g., microbes, infections, etc.), antitumor resistance, and pro-inflammatory cytokines [152]. On the other hand, angiogenesis, tumor growth, and immunosuppression are all aided by activated M2 macrophages. Treg and T-helper (Th) 2 cell recruitment is aided by macrophage M2. (Th) 2 cells secrete cytokines including IL-4, IL-5, and IL-10, while Treg cells suppress the immune system, which ultimately promotes the formation of tumors [153]. TAMs are further subdivided into four macrophage subtypes, M2a, M2b, M2c, and M2d, based on different stimuli. M2a macrophages are engaged in allergies and parasite elimination and are stimulated by IL-13 and IL-4, two cytokines. M2b Among macrophages is crucial for Th 2 cell activation and immunoregulation. They are activated by attaching agonists to IL or TLRs. IL-10 activates M2 macrophages, which are in charge of immunoregulation, matrix deposition, and tissue remodeling. TLR agonists and IL-6 have recently been shown to activate a fourth subtype of alternative M2d macrophages through adenosine receptors. Anti-inflammatory cytokines (like IL-10 and IL-12) and angiogenic factors (like VEGF) are produced when adenosine receptors are activated. The critical function that TAMs play in tumor maintenance, development, and metastasis is supported by these investigations [154].

#### 1.1.13 Function of tumor-associated neutrophils in the development and spread of gliomas

Another class of cellular constituents that are involved in human defense against pathogen-induced infection and inflammation resolution is neutrophils [155]. While neutrophils are mostly involved in tissue homeostasis and host defense, they may also contribute to long-term inflammation and the development of cancer in various diseases [156]. In the initial stages of inflammation or injury, chemotactic agents like GM-CSF, IL-6, IL-8, and CXCL1 attract neutrophils to the lesion site where they secrete IL-1β, TNF-α, IL-6, IL-12, MMP-9, VEGF, and arginase-1. These factors ultimately cause chronic inflammation, promote angiogenesis, and induce an immunosuppressive state [157]. Neutrophils have a longer lifespan in inflammation because pro-inflammatory mediators such as LPS, G-CSF, and IL-2 prevent them from going through programmed cell death (apoptosis) [152]. The N1 phenotype, which is pro-inflammatory and anti-tumorigenic, and the N2 phenotype, which is pro-tumorigenic, are further subclassifications of tumor-associated neutrophils (TANs) [158]. TGF-β can polarize N1 neutrophils to a N2 phenotype, whereas IFN-β can polarize N2 neutrophils to a N1 phenotype. N1 neutrophils perform a variety of biological tasks, including cytotoxicity against tumor cells, T-cell activation, and tumorigenesis prevention. On the other hand, N2 neutrophils support angiogenesis, immunological suppression, tumor invasion, and cancer cell stemness maintenance [159]. Neutrophils (about 50 %) and platelets (about 25 %) have high levels of circulating VEGF, a crucial regulator of endothelial cell activity and angiogenesis [160]. As a tumor grows, the N1 type of TAN activity is downregulated and most take on the N2 phenotype [161]. The rate of TAN infiltration is higher in high-grade human GBM than in low-grade GBM. A study using a mouse model of carcinogenesis showed that proinflammatory IL-17-mediated CXCR2 axis activation results in a high population of neutrophils at the tumor site [162]. In GBM patients, elevated levels of the interleukin IL-12 subtype (IL-12p70) levels were associated with increased neutrophil activity. In these patients, neutrophil actuation was found to be an early indicator of tumor movement [163]. Research has shown that in patients with GBM, increased neutrophil degranulation is associated with immunosuppression and is associated with higher levels of arginase1 (Arg-1) [164]. Macrophages phagocytose neutrophils after an infection or inflammation has been treated. Moreover, the immunosuppressive cascade is initiated by the death of recruited neutrophils, just as it occurs in the tissue repair mechanism that is mostly carried out by type M2 macrophages [165].

#### 1.1.14 The connection between TAMs and inflammatory mediators

**Fig. 4:**
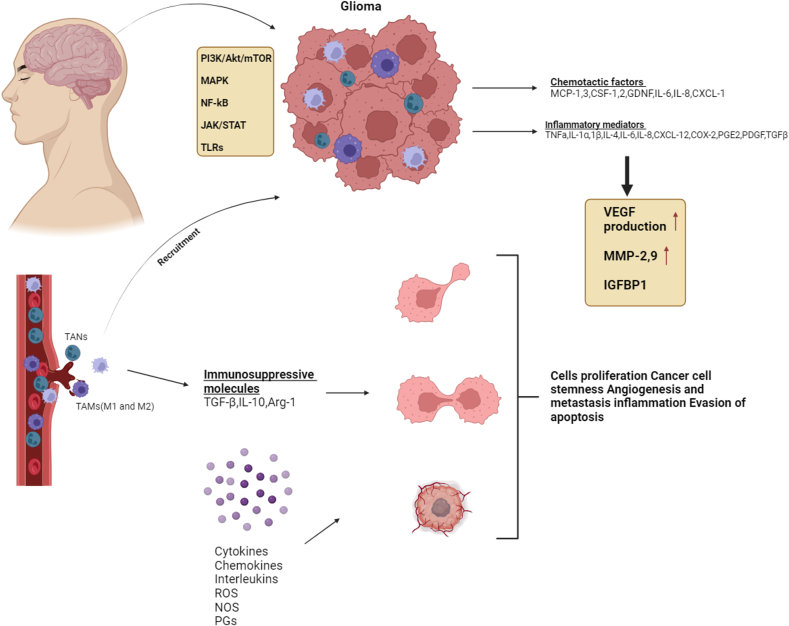
The involvement of signaling pathways, TAMs, TANs, and inflammatory mediators in the tumorigenicity of gliomas. Activation/release is indicated by the color green. Tumor-associated neutrophils (TANs) and macrophages (TAMs) CSF-1,2 and MCP-1,3 are colony-stimulating factors and monocyte chemoattractant proteins, respectively. The cell-derived neurotrophic factor is commonly referred to as GDNF. Various interleukins such as IL-1 α, β, −4, −6, −8, −10, and chemokines like CXCL-1, -12 are involved in immune responses. Additionally, TNF-α, COX-2, prostaglandin E2, PDGF, TGF β, VEGF, MMP-2, 9, IGFBP1, and Bmi-1 represent different signaling molecules and growth factors. Arg-1 denotes arginase-1, NF-κB stands for nuclear factor kappa light chain enhancer of activated B cells, and TLRs refer to toll-like receptors. Furthermore, JAK/STAT denotes Janus kinase-signal transducer and activator of transcription, MAPK represents mitogen-activated protein kinase, and PI3K/Akt/mTOR denotes phosphoinositol-3-kinase-mammalian target of rapamycin.

In the glioma microenvironment, there is a high presence of inflammatory mediators and chemotactic substances, including colony-stimulating figure (CSF-1, -2) and glial cell-derived neurotrophic figure (GDNF). These factors are responsible for attracting TAMs to the tumor site and polarizing TAMs from M1 to M2 phenotypes, which ultimately triggers the tumor-initiating process [166,167]. An increased number of TAMs in GBM tissue samples has been linked to high IL-6 levels in cerebrospinal fluid [143]. The first chemotactic factor was found to be monocyte chemo-attractant protein-1, which is secreted by a variety of cells, including the endothelium, fibroblasts, epithelial, astrocytic, monocytic, and microglial cells [168]. Notably, in the rat glioma model, a substantial association was found between invading microglial cells and elevated production of either MCP-1 or CCL2. When comparing GBM samples to control samples, it was shown that the microglial cell population had increased by almost 10 times [169]. Similarly, in human GBM cells, the release of MCP-3 generated from gliomas aided in the recruitment of TAMs [170]. The glial cell-derived neurotrophic figure (GDNF) and the changing development factor-β superfamily display outstanding basic likeness. Research findings have revealed the similarity between these growth factors, which were initially discovered in the B49 rat glioma cell line, which is in charge of the survival and development of neural cells in the central nervous system, including dopaminergic neurons [171]. Using its interaction with its cognate receptor, GDNF receptor-α1, GDNF directly contributes to the growth of gliomas [172]. Moreover, a human glioblastoma mass was found to have GDNF levels that were almost five times higher than those of normal brain tissue [172]. The GDNF released by gliomas in humans and rodents acts as a potent chemotactic factor for brain-resident macrophages or, microglia. According to a different study, glioma cells with hollow fibers around them produce only a range of soluble components. In the mouse brain, neighboring microglial cells are drawn to and gather around these hollow fibers as a result of the tumor cells' production of GDNF [173]. Glioma progression and microglial activation are induced by tumor-derived macrophage colony-stimulating factor (M-CSF, CSF1) and granulocyte-macrophage colony-stimulating factor (GM-CSF, CSF2) [174]. Additionally, These components have been shown to interact with other chemicals in the body that cause inflammation, like IL-1 and TNF [175]. Angiogenesis in several cancer types is linked to increased M-CSF/CSF1 expression. Microglial cells were encouraged to secrete insulin-like growth factor-binding protein 1 (IGFBP1) to promote angiogenesis in GBM-derived M-CSF [176]. In human gliomas, CSF2/GM-CSF stimulated microglia to increase the ability of tumor cells to infiltrate [174]. Another study showed that the presence of CSF2/GM-CSF increases the migration ability of glioma cells and increases their resistance to apoptosis [177]. The question of whether TAMs simply observe or actively engage in the invasion and evolution of gliomas is raised by their recruitment. There is strong evidence that TAMs actively participate in the genesis of gliomas. Microglial cells were found to have a threefold effect on the movement of mouse-derived glioma cells [178]. In vitro research using murine BV2 microglial cells demonstrated that conditioned media from glioma cells might activate microglial cells by increasing the mRNA levels of cyclooxygenase-2 (COX-2), tumor TNF-α, IL-6, iNOS, and IL-1β. Tumor is a word used to describe a certain type of growth in the body. TNF-α, IL-6, iNOS, and IL-1β are names of substances or molecules that can be found in the body. By activating NF-κB and p38 MAPK, activation, enhanced tumor development [179]. It was discovered that glioblastoma progression is actively influenced by a few soluble proteins generated from microglia, including transforming growth factor-β (TGF-β), epidermal growth factor (EGF), and stress-inducible protein 1 (STI 1) [94]. Infiltrated TAMs and glioma tissue both in vitro and in vivo showed elevated expression of the cellular prion protein STI 1, which was positively associated with higher tumor growth [180]. When EGFR is turned on in human GBM cells, a protein called VCAM-1 becomes more active. This helps tumor cells and immune cells called macrophages communicate better, which helps the tumor invade other parts of the body [181]. AHR, which is activated by kynurenine generated by GBM cells, plays a crucial role in TAM function and cell immunity, according to a recent study. By increasing adenosine in conjunction with CD73, AHR facilitates CCR2 expression-mediated TAM recruitment and ectonucleotidase CD39-mediated CD8^+^ T cell malfunction. Based on these results, AHR and CD39 could be good targets for improving the body's defense against GBM and other types of cancer [182]. TGF-β is a cytokine that is both tumor-suppressive and tumor-promoting, and it is significantly expressed in brain tumors. Gliomas are among the various tumor types that are promoted to grow when there is a mutation in the TGF-β signaling pathway [183]. When glioma cells were cocultured with rat primary microglial cells, glioma invasion was observed, resulting in approximately a 200-fold rise in TGF-β1. The result was made easier by activating the TGF-β type II receptor (TβIIR) [184]. In LN-229 glioma cells, IL-1β and TGF-β made the cells form neurosphere and increased the presence of Bmi-1 and nestin genes related to tumor stemness [185]. In the early phases of tumor formation, tumor-bearing animals expressed more M1 macrophages than M2 macrophages; in contrast, the M2 phenotype was observed in the later stages of cancer [186]. When TAMs change to the M2 state, they produce substances such as TGF-β, EGF, MMP-2, MMP-9, and IL-10. These substances help tumors grow blood vessels, spread, and create an environment where the immune system fails to work properly [187]. Furthermore, a positive correlation was found between the glioma grade and the levels of M2 state markers, such as CD14 and CD68 [188]. The M2 phenotype of macrophages secreted IL-1β, which led to the migration of glioma cells. Subsequently, IL-1β stimulates the phosphatidylinositol-3-kinase (PI3K) pathway and phosphorylates the glycolytic enzyme glycerol-3-phosphate dehydrogenase (GPD3), thereby promoting the survival and proliferation of tumor cells [189]. One thing that is different about cancer working in the body is that it weakens the immune system. The NF-κB pathway must start working in GBM because of the presence of TAM polarization and immune suppression [156]. Studies have found that GM-CSF makes TAMs move toward it and boosts the ability of certain immune cells to suppress the immune system by turning on the IL-4Rα [190]. These findings support the significance of TAMs as a possible target for the treatment of GBM and other cancers and point to their pivotal role in the initiation and dissemination of the disease (Fig. 4).

#### 1.1.15 Antibody checkpoints

**Fig. 5:**
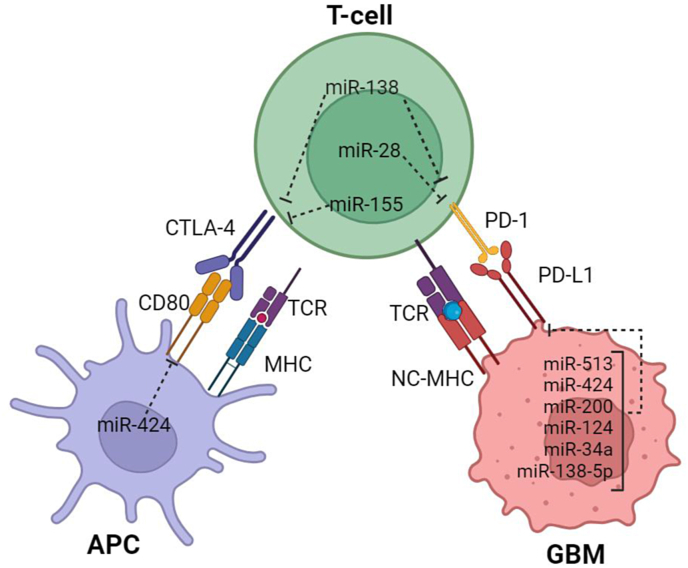
APC, T cells, and GBM interact to activate immunological checkpoints that allow the GBM to escape the immune system. The illustration illustrates how miRNA molecules are beginning to exert inhibitory effects on the indicated axis. MiR-138 on T cells interact with PD-1 and CTLA-4, leading to their inhibition. In addition, miR-28 and miR-155 are involved in the suppression of PD-1 and CTLA-4, respectively.

MirRNA-targeting molecules known as Immune Checkpoint (IC) present new avenues for controlling GBM, as recent investigations have demonstrated. In patients with advanced melanoma, ipilimumab is the first drug to block the CTLA-4 protein, which helps the immune system fight cancer. By attaching to CD 86 and CD 80 on antigen-presenting cells, CTLA-4 prevents appropriate T cell co-stimulatory signaling. There exists an inverse relationship between the ultimate result in GBM patients and the molecules expressed on their CD8^+^ and CD4^+^ cells [191,192]. As part of the IC family, PD-L1 is important for controlling the immune system and glioma cells respond to it. T-cell cytotoxicity and proliferation are significantly reduced upon activation of the PD-1 receptor. Additional signaling promotes immune escape and tumor evasion. The stimulation of several receptors, including the toll-like receptors (TLRs), intergalactic receptors (IFNAR, IFNGR), and epidermal growth factor receptor (EGFR), occurs before the overexpression of PD-L1. Several pathways, including the phosphatase and tensin homolog deleted on chromosome ten/the mammalian target of rapamycin (PTEN/mTOR), signal transducer and activator of transcription 3/mitogen-activated protein kinase/extracellular signal-regulated kinases (STAT3/MEK/ERK), or Janus-activated kinases/signal transducers and activators of transcription (JAK/STAT), are used to down-signalize self-activated glioma cells. These pathways facilitate the transcription, translation, and surface presentation of PD-1L mRNA [193]. MicroRNA molecules have the power to influence the processes mentioned above. Wang et al. examined 49 miRNA particles that control the interaction between PD-L1 and PD-1 in patients with cancer. IDH 1/2 mutations and the immunological diversity of GBM are complementary. Heiland et al. verified that IDH1/2 wild-type cells had upregulated PD-L1 expression in contrast to IDH1/2 mutant cells, which have reduced levels of PD-L1 expression due to hypermethylation of the PD-L1 promoter region. These results may explain the different reactions to PD-L1 inhibition in patients who met recruitment trial eligibility requirements and identify molecules like miRNAs that interact with ICs at the epigenetic level. In addition, miRNAs block the CTLA-4 and PD-1/PDL1 paths. Certain molecules like miR-28, miR-34a, mir-124, mir-138, miR-138-5p, mir-155, miR-200, miR-424, and miR-513 interact with specific ICs [192]. Additionally, miRNAs may indirectly interact with ICs. Some regulate transcription and trigger IFN gamma release, forming intricate relationships. Research on MiR-34a has shown that it can affect the expression of PD-L1 and epidermal growth factor receptor (EGFR) to have an antitumor effect on glioma cells. By suppressing FKBP51, a PD-L1 cochaperone molecule, MiR-34a prevents PD-L1 maturation. In this way, the PD-L1/PD-1 axis-induced resistance to therapy can be reduced. Better results were correlated with miR-34a expression [194,195]. Agreeing with Chen et al.’s epigenetic inquiry, the expression of PD-L1 and the miR-200 molecule are contradictory [196]. The same relationship between Pd-L1 and miR-138-5p was reported by Wei et al. His research indicated that MiR-138 also decreased expression on the surface of CD4^+^ T cells and targeted PD-1 and CTLA-4. Research conducted using mouse models revealed a considerable extension of the median survival period and tumor mass regression. Tumor regression persisted following the cessation of miR-138 ingestion [197]. Moreover, miR-124 smothers the flag transducer and the activator of translation 3 (STAT3), which in turn decreases the expression of immune-suppressive particles like PD-1. In all types of malignant tumors, including high-grade gliomas, the expression of MiR-124 is restricted. Glioma cancer stem cells (gCSC) that have been immunosuppressive modified are impacted by miR-124. To preserve immune escape, systemic management modifies the glioma microenvironment and strengthens the anticancer characteristics [198]. The development of local inflammation is facilitated by the upregulation of miR-155, which results in the initiation of CTLA-4, which subsequently causes tumor invasion [199]. Replication processes controlled by Mir-28 decrease PD-1 expression when it attaches to the 3′UTR. By altering cell cycles, T lymphocytes are depleted [200]. In gliomas, miR-424 and miR-513 prevent the production of PD-1L. Furthermore, miR 424 suppresses the CD 80 molecule on antigen-presenting cells (APCs), which indirectly reduces CTLA-4 signaling [201]. Moreover, miRNAs interfere with the CTLA-4 and PD-1/PDL1 pathways. Certain molecules like miR-28, miR-34a, miR-124, miR-138, miR-138-5p, miR-155, miR-200, miR-424, and miR-513 interact with specific immune system cells [202]. Additionally, miRNAs may indirectly interact with IC. Some regulate transcription and trigger IFN gamma release, forming intricate relationships. Research has shown that MiR-34a can affect PD-L1 and EGFR expression, which can help stop cancer cell growth in the brain. By suppressing FKBP51, a PD-L1 cochaperone molecule, MiR-34a prevents PD-L1 maturation. In this way, the PD-L1/PD-1 axis-induced resistance to therapy can be reduced. Better results were correlated with miR-34a expression [194]. Chen et al.’s epigenetic research determined that the miR200 particle has a reciprocal relationship with PD-L1 expression [196]. The same relationship between Pd-L1 and miR-138-5p was reported by Wei et al. This investigation showed that MiR-138 decreased expression on the surface of CD4^+^ T cells and targeted PD1 and CTLA-4. Research conducted using mouse models revealed a considerable extension of the median survival period and a regression of the tumor mass (Fig. 5). Tumor regression persisted following cessation of miR-138 ingestion [203]. Furthermore, miR-124 suppresses signal transducer and transcription activator 3 (STAT3) signaling, resulting in the downregulation of immune-suppressive molecules like PD-1. In all types of malignant tumors, including high-grade gliomas, the expression of MiR-124 is restricted. Glioma cancer stem cells (gCSC) that have been immunosuppressive modified are impacted by miR-124. To preserve immune escape, systemic management modifies the glioma microenvironment and strengthens the anticancer characteristics [198]. The development of local inflammation is facilitated by the upregulation of miR-155, which results in the initiation of CTLA-4, which in turn causes tumor invasion [199]. Replication processes controlled by Mir-28 decrease PD-1 expression when it attaches to the 3 UTR. Altering the cell cycle T lymphocytes [204]. In gliomas, miR-424 and miR-513 suppress PD-1L production. The CD 80 molecule in antigen-presenting cells (APCs) is also inhibited by miR 424, which indirectly suppresses CTLA-4 signaling [205].

In GBM, certain small RNA molecules like miR-513, miR-424, miR-200, miR-138-5p, miR-124, and miR-34a stop PD-1L. Additionally, miR-424 stops the CD80 molecule from being produced by cells that present antigens, which is important for activating the CTLA-4 receptor in T cells. Big collection of genes that help the immune system, another collection of genes that help the immune system but are different, a receptor on T cells, a protein on T cells, a protein that can stop immune responses, a protein that helps immune cells communicate, and a protein that can stop immune responses. MiR-513 is also reduced by IFN, which decreases its ability to inhibit PD-1L expression in GBM cells. More research is needed to understand how miRNAs and ICs interact because even though the results from testing on animals are promising, they are still not fully understood.

#### 1.1.16 Main microRNAs as diagnostic biomarkers of GBM

Given the challenges associated with imaging diagnosis, monitoring the biochemical marker levels is crucial in patients with GMB. The treatment being delivered causes the tumor cells to absorb more contrast material, which contaminates radiosensitive image capture and makes therapy control much more difficult [206]. This is why finding new markers, like microRNAs, requires significant work. Alzheimer's disease, along with various types of cancer, including papillary thyroid carcinoma, breast cancer, gastric cancer metastasis, Hodgkin lymphoma, and pancreatic ductal adenocarcinoma, has been associated with the importance of microRNA (miRNA) as a diagnostic marker for these diverse pathologies [[207], [208], [209]]. Using astrocytoma samples, an initial investigation into the significance of miRNA in the diagnosis of gliomas was conducted. Zhi et al. identified seven miRNA particles, whose amounts differed markedly from brain tissue in healthy individuals [210]. Numerous additional studies were also conducted, one of which focused on high-grade gliomas [211]. Chen et al.’s observations revealed seven more miRNA examples that might be useful as markers. MiR7, miR15b, miR21, miR124a, miR129, miR139, and miR218 comprised the group [212]. Srinivasan et al. conducted a comprehensive meta-analysis highlighting the centrality of 10 miRNAs that can be used to estimate the course of malady in patients with GBM. Furthermore, many of them were linked to a worsening disease course, while others suggested a favorable prognosis. MiR31, miR146b, miR148a, miR193a, miR200b, and miR221 were among the members of the first group [213]. Papillary thyroid carcinoma and squamous cell lung cancer have both shown comparable connections with miR146b [214]. It's interesting to note that several researchers have stressed the role this molecule plays in blocking metastatic alterations [215,216]. MiR31 and breast cancer have been linked in similar ways [217]. Likewise, other studies have validated the function of miR221 in the etiology of malignancies, including GBM. The roles of particles p27, p53, PTEN, and p27Kip1 in this process have received special attention [218,219]. Regarding melanoma, associations with poor prognosis were identified in patients with miR193a [220]. Srinivasan et al. mentioned miR17-5p, miR20a, and miR106a as potential protective compounds [221]. Other studies on colorectal cancer and glioblastomas have identified a similar link to miR106a [212,222]. However, the carcinogenic potential of this article has been demonstrated in the context of T-cell leukemia [223]. Studies examining the objectives of these particles (cyclin D1, E2F1) have shown similar results regarding the good prognostic relevance of miR17 and miR20a (together included in the miR17-92 cluster) in gliomas [224,225]. Furthermore, studies conducted on a variety of cancer types revealed that they can act as both oncogenes and suppressors within the cell [226,227]. Numerous investigators have also identified other miRNAs, the presence of which may be linked to improved prognosis and extended survival in patients with glioma and glioblastoma. This indicates that they can be employed as biomarkers of disease progression. Table 2 lists miR29c, miR101, miR107, miR144-3p, mir181d, miR203, miR205, and miR328 as members of this group [[228], [229], [230]].

**Table 2:** Specific mRNAs with different expressions in Glioblastoma Multiforme. mi

Decreased	Decreased more10x	Decreased 2-10x	Fold	Increased	Increased 2-10x	Increased more10x
miR106a miR181a miR181b miR448 miR490 miR876-3p	miR33 miR105 miR124a miR124b miR128a miR128b miR129 miR132 miR133a miR133b miR137 miR139 miR153 miR154 miR184 miR203 miR218 miR326 miR330 miR338	miR29cN miR31 miR95 miR107 miR122a miR149 miR154 miR190 miR219 miR221 miR299 miR321 miR323 miR328 miR331 miR340 miR342 miR370	miR30a-3p miR143 miR186 miR324-3p miR335 miR337	miR26a miR221/222 miR335 miR451 miR486 miR501-3p	miR15b miR17-5p miR21 miR23a miR25 miR104 miR106b miR124 miR148a miR182 miR183 miR188 miR199a miR199b miR199s miR200cN miR210 N miR224 miR268 miR273	miR10a miR10b miR96

Qiu et al. performed thorough research on 480 GMB samples and observed that patients with higher survival rates had low miR155, miR210, miR323, and miR329 levels and high miR130a and miR326, respectively. Furthermore, progression-free survival (PFS) was associated with miR155, miR210, miR130a, and miR326 [231]. These data continue to support Schiesser et al.’s observations. Low miR155 and miR210 levels were associated with a worse prognosis. Only miR155 was a predictive factor independent of other patient variables, such as MGMT methylation, IDH1/IDH2 mutation, and histology, according to their investigation of 29 miRNA locations with different levels of methylation [232]. The degree of tumor histological staging is frequently linked to the presence of certain miRNAs and a patient's life expectancy. It was demonstrated in the work of Regazzo et al. that determining the expression level of certain miRNAs (miR125b, miR497) can reveal whether the tumor under examination is a low-grade or high-grade glioma [233]. Wang et al. reached similar conclusions, reporting that individuals with gliomas had decreased levels of miR342-3p in their serum [234]. However, the extent of this decrease varies depending on the grade of the tumor. A few other studies have shown similar connections, like those involving miR19, miR137, miR144-3p, and miR182 [[227], [228], [229]]. Additionally, it was shown that le7e, miR145, and miR181a had a negative correlation with the tumor histology stage, while miR19 and miR182 had a favorable correlation [235]. This would allow for improved procedure planning by offering crucial guidance on the tumor's features before surgery and the slice's histological analysis. Thus, tracking the levels of miRNA markers could help identify the transition of low-grade gliomas into high-grade gliomas. Malkorn et al. studied WHO grade II gliomas that developed into WHO grade IV GBM. Several markers that aid in identifying the stage of histological development were indicated by these markers. These comprised the following: miR210 (elevated level); miR184 and miR328 (reduced level); and miR9, miR15a, miR16, miR17, miR19a, miR20a, miR21, miR25, miR28, miR130b, and miR140 [236]. Wang et al. also discovered that anti-cancer treatment alters the levels of miR21, miR128, and miR342-3p, which then recover to levels typical of healthy tissue [234]. Similar findings were also observed in other studies on miR128 [237] for miR128. This suggests that miRNAs can be used as indicators of treatment efficacy. Importantly, the performance of microRNAs will differ depending on the stimulation and effect of different drugs [238]. Therefore, the effect of combination therapy, biochemicals, and different enzymes is of great importance in GBM therapy [231].

## 2 The role of the microbiome in glioblastoma

### 2.1 Gut microbiota and immune system modulation

The bacteria in the gastrointestinal tract can significantly impact the effectiveness of certain cancer treatments, particularly immune checkpoint inhibitors (ICIs) used to treat glioblastoma. Microbial byproducts, such as short-chain fatty acids, can influence immune system function by altering how immune cells behave and where they are directed. This, in turn, can increase or decrease the inflammation around tumors. A healthy balance of gastrointestinal bacteria may improve the efficacy of ICIs for treating glioblastoma. Gut bacteria can also affect biological processes that influence the growth of glioblastoma, a type of brain cancer. For instance, substances produced by these microbes can modify gene expression in tumor cells, thereby affecting their behavior. This can impact the formation of new blood vessels, the infiltration of immune cells into the tumor, and the overall characteristics of the tumor itself. These interactions illustrate how signals from the gut can affect the tumor microenvironment and contribute to the development of glioblastoma. Additionally, the gut-brain connection plays a vital role in the influence of gut bacteria on the health of the brain and nervous system. Changes in gastrointestinal bacteria can affect the brain's immune system and the activity of immune cells involved in glioblastoma growth [239,240].

### 2.2 Host-microbial interactions and TLR signaling

#### 2.2.1 TLRs as microbial signal sensors

Toll-like receptors are crucial components of the body's first line of defense against germs. They help the immune system detect harmful microorganisms, distinguish between helpful and harmful germs, and initiate the necessary immune responses to maintain balance or trigger inflammation. In the context of glioblastoma, changes in the types of microbe's present can influence TLR signaling, potentially altering the immune response in the tumor environment. Activating TLRs can affect cytokine production and immune cell function, which may either promote or inhibit tumor growth. For instance, activated TLRs enhance the body's ability to fight cancer by improving the function of dendritic cells and facilitating T-cell activation. However, if TLR activation is not properly regulated, it can induce conditions that weaken the immune response. This finding highlights the importance of understanding how microbes affect TLR signaling in glioblastoma. Gut bacteria play a critical role in regulating the immune system; thus, researchers are investigating fecal microbiota transplantation (FMT) as a strategy to enhance the effectiveness of immunotherapy. By restoring a healthy balance of microorganisms in the body, FMT may improve patient responses to immunotherapy. This approach underscores the necessity of personalized treatment plans that account for each individual's unique microbiome. Gaining insights into how specific microbes interact with TLR signaling pathways could lead to new treatment strategies and better patient outcomes [241].

## 3 Conclusion

GBM is a serious malignant brain tumor that is extremely invasive, resistant to existing treatments, and persistently grows. The prognosis of patients with gliomas remains bleak, even with recent breakthroughs in therapy. In the immune system, TLRs are the first line of defense. GBM, especially HGG, is seen as a tough tumor because it spreads quickly, has short survival rates, often comes back, and does not respond well to usual treatments. Glioma immunotherapy can be enhanced by TLR signaling and its wide range of interactions. As illustrated, a focus on TLR signaling can influence a wide extent of forms. Through the utilization of TLR agonists to induce PD-1/PD-L1 expression, TLR-expressing GSCs and resistant cells are balanced to create an antitumor microenvironment, and suppress, pro-tumorigenic signaling and undesirable GAM aggregation. Although encouraging outcomes have been noted, more clinical observations are necessary to address the present shortcomings and develop the most effective treatment plans that can give glioma patients fresh hope. In the future, we hope that TLR-based therapies will yield encouraging outcomes for the effective treatment of gliomas.

## 4 Strengths and limitations of this research

### 4.1 Strengths

This study highlights two key functions of TLRs in GBM. TLRs can activate both tumor-fighting responses and actions that promote tumor growth. This dual role presents new opportunities for immunotherapy and could enhance treatment effectiveness when TLRs are precisely targeted. TLRs are promising candidates for immunotherapy because they can initiate a response. Activating the TLR pathway helps dendritic cells mature and present antigens more effectively, which is crucial for a robust anticancer immune response. This indicates that TLR agonists can improve patient outcomes when used in combination with other treatments, such as chemotherapy and checkpoint inhibitors. Numerous studies have demonstrated that TLR agonists can help patients with GBM live longer. Recent research on combination therapies involving TLRs is essential for developing strategies to prevent treatment resistance and enhance patient outcomes. This study provides important insights into how TLR signaling interacts with the tumor microenvironment and the surrounding immune system, thereby improving our understanding of tumor growth and evasion in glioblastoma.

## 5 Limitations

The signaling pathways triggered by TLRs are complex and can produce different effects depending on factors such as the type of ligand, surrounding cells, and the presence of other signaling molecules. This complexity makes it challenging to predict how individuals will respond to treatment. While TLR activation can enhance immune responses, it may also promote tumor growth by causing proliferation and migration of cells. This dual role complicates the use of TLR agonists in treatment because their activation can inadvertently support tumor growth. Glioblastoma exhibits significant variability in its molecular composition, both among different tumors and within various regions of the same tumor. Variations in patient responses can lead to different reactions to TLR-targeted treatments, making it difficult to devise a one-size-fits-all treatment plan. Although there is increasing evidence that TLRs play a role in GBM, we still do not fully understand how they function in tumor and immune cells. More research is necessary to gain a better understanding of these processes and to enhance treatment strategies.

## CRediT authorship contribution statement

**Seyedeh Elham Norollahi:** Writing – original draft, Methodology, Investigation. **Kosar Babaei:** Writing – original draft, Investigation. **Ali Rashidy-pour:** Methodology, Investigation. **Bahman Yousefi:** Writing – original draft, Investigation. **Rasoul Baharlou:** Writing – original draft, Investigation. **Bahareh Farasati Far:** Writing – original draft, Investigation. **Amir Jalali:** Writing – original draft, Investigation. **Ali Akbar Samadani:** Writing – review & editing, Validation, Methodology, Investigation, Conceptualization.

## Funding

It is a narrative review article.

## Declaration of competing interest

Hope you are doing well. According to the submission of our manuscript which is entitled: **“Role of TLR signaling pathway in the pathogenesis of glioblastoma multiforme with emphasis on immunotherapy”** In this journal, all authors declare that there is no conflict of interest and also all the ethical standards considered carefully. Remarkably, all the authors studied and confirmed the final edited version of this manuscript.

We hope that the manuscript will receive a fair review and will hear from you positively soon.

## Data Availability

The data that has been used is confidential.

## Abbreviations

**APC**: Antigen-presenting cell
**AMPK**: Adenosine monophosphate-activated protein kinase
**Arg-1**: Arginase1
**BBB**: Blood-brain barrier
**BMDM**: Bone marrow-derived macrophages
**CTLA**: Cytotoxic T-lymphocyte antigen
**CNT**: Carbon nanotube
**CAR**: Chimeric antigen receptor
**CNS**: Central nervous system
**CpG-ODN**: Cytosine–phosphate–guanosine oligodeoxynucleotide
**CPS**: Complex polysaccharides
**CSF-1**: Colony-stimulating factor1
**CCL**: C–C motif chemokine ligand
**CXCL**: Chemokine (C-X-C motif) ligand
**COX2**: Cyclooxygenase-2
**DAMP**: Damage-associated molecular patterns
**DC**: Dendritic cell
**EMA**: Epithelial membrane antigen
**EGFR**: Epidermal growth factor receptor
**ERK**: Extracellular signal-regulated kinase
**FDA**: Food and Drug Administration
**GBM**: Glioblastoma multiforme
**GFAP**: Glial fibrillary acidic protein
**GDNF**: Glial cell-derived neurotrophic factor
**GPD3**: Glycerol-3-phosphate dehydrogenase
**gCSC**: Glioma cancer stem cells
**HMGB**: High-mobility group box protein
**HSP**: Heat shock protein
**HPC**: Hepatic progenitor cell
**IFN**: Interferon
**IC**: Immune checkpoint
**IgG**: Immunoglobulin G
**ITSM**: Immunoreceptor tyrosine-based switch motif
**IL**: Interleukin
**IDO**: Indoleamine 2,3-dioxygenase
**ICPI**: Immune checkpoint inhibitor
**IGFBP1**: Insulin-like growth factor-binding protein 1
**JNK**: C-Jun kinase
**LPS**: Lipopolysaccharide
**LAG**: lymphocyte activation gene
**MAMP**: Microbe-associated molecular patterns
**mAbs**: Monoclonal antibodies
**MAPK**: mitogen-activated protein kinase
**mDC**: Myeloid dendritic cell
**MDSC**: Myeloid-derived suppressor cell
**MMP**: Matrix metalloproteinase
**mTOR**: Mammalian target of rapamycin
**MT1-MMP**: Membrane type 1 matrix metalloprotease
**MyD88**: Myeloid differentiation factor 88
**MHC**: Major histocompatibility complex
**NK**: Natural killer cells
**NF-κB**: Nuclear factor kappa B
**NC-MHC**: Non-Classical Major Histocompatibility Complex
**PD-1**: Programmed cell death protein 1
**PD-L1**: Programmed death Ligand 1
**PAMP**: Pathogen-associated molecular pattern
**PGE-2**: Prostaglandin E2
**PI3K**: Phosphoinositide 3-kinase
**pDC**: Plasmacytoid dendritic cell
**PFS**: Progression-free survival
**Poly ICLC**: Polyinosinic–polycytidylic acid, and poly-l-lysine
**PG**: Prostaglandin
**PDGF**: Platelet-derived growth factor
**PTEN**: Phosphatase and tensin homolog on chromosome 10
**RAGE**: Receptor for advanced glycation end products
**RIP**: Receptor-interacting serine/threonine protein
**STAT3**: Signal transducer and activator of transcription 3
**TLR**: Toll-like receptor
**TCR**: T Cell Receptor
**Th**: T-helper cell
**TME**: Tumor microenvironment
**TMZ**: Temozolomide
**TK**: Thymidine kinase
**TNF**: Tumor necrosis factor
**TRAF**: Tumor necrosis factor receptor-associated factor
**TGF-β**: Transforming growth factor-β
**Treg**: Regulatory T-cell
**TAMs**: Tumor-associated macrophages
**TIM-3**: T cell immunoglobulin mucin 3
**TAN**: Tumor-associated neutrophils
**TβIIR**: TGF-β type II receptor
**VEGF**: Vascular endothelial growth factor
**VCAM-1**: Vascular cell adhesion molecule-1
**WHO**: World Health Organization
