# Supplementary material for: Role of the TLR signaling pathway in the pathogenesis of glioblastoma multiforme with an emphasis on immunotherapy
Source: Biochem Biophys Rep. 2025 Jul 18;43:102149. doi: 10.1016/j.bbrep.2025.102149 (PMC12302769; doi:10.1016/j.bbrep.2025.102149)
Supplement: Multimedia component 1 [file mmc1.docx]

**Dear Editor,**

Hope you are doing well. By the way, I submitted this manuscript which is entitled: **The role of TLR signaling pathway and cellular and molecular factors involved in the pathogenesis of glioblastoma multiforme with emphasis on immunotherapy** in this journal, and request a full waiver for APC. Unfortunately, because of the hard and serious sanctions of the USA on my country, we are not able to transfer the money. I will be so thankful if you consider my request. Thank you so much for your deep understanding and attention.

With best and warm regards

**Ali Akbar Samadani, PhD,** Guilan Road Trauma Research Center, Trauma Institute, Guilan University of Medical Sciences, Rasht, Iran

Email:a.a.hormoz@gmail.com
